# First person – Jun-yi Zhu

**DOI:** 10.1242/dmm.050591

**Published:** 2023-12-27

## Abstract

First Person is a series of interviews with the first authors of a selection of papers published in Disease Models & Mechanisms, helping researchers promote themselves alongside their papers. Jun-yi Zhu is first author on ‘ *APOL1-G2* accelerates nephrocyte cell death by inhibiting the autophagy pathway’, published in DMM. Jun-yi is an Assistant professor in the lab of Zhe Han at the University of Maryland School of Medicine, Baltimore, USA, investigating *Drosophila* as a model to study human disease mechanisms and treatment approaches.


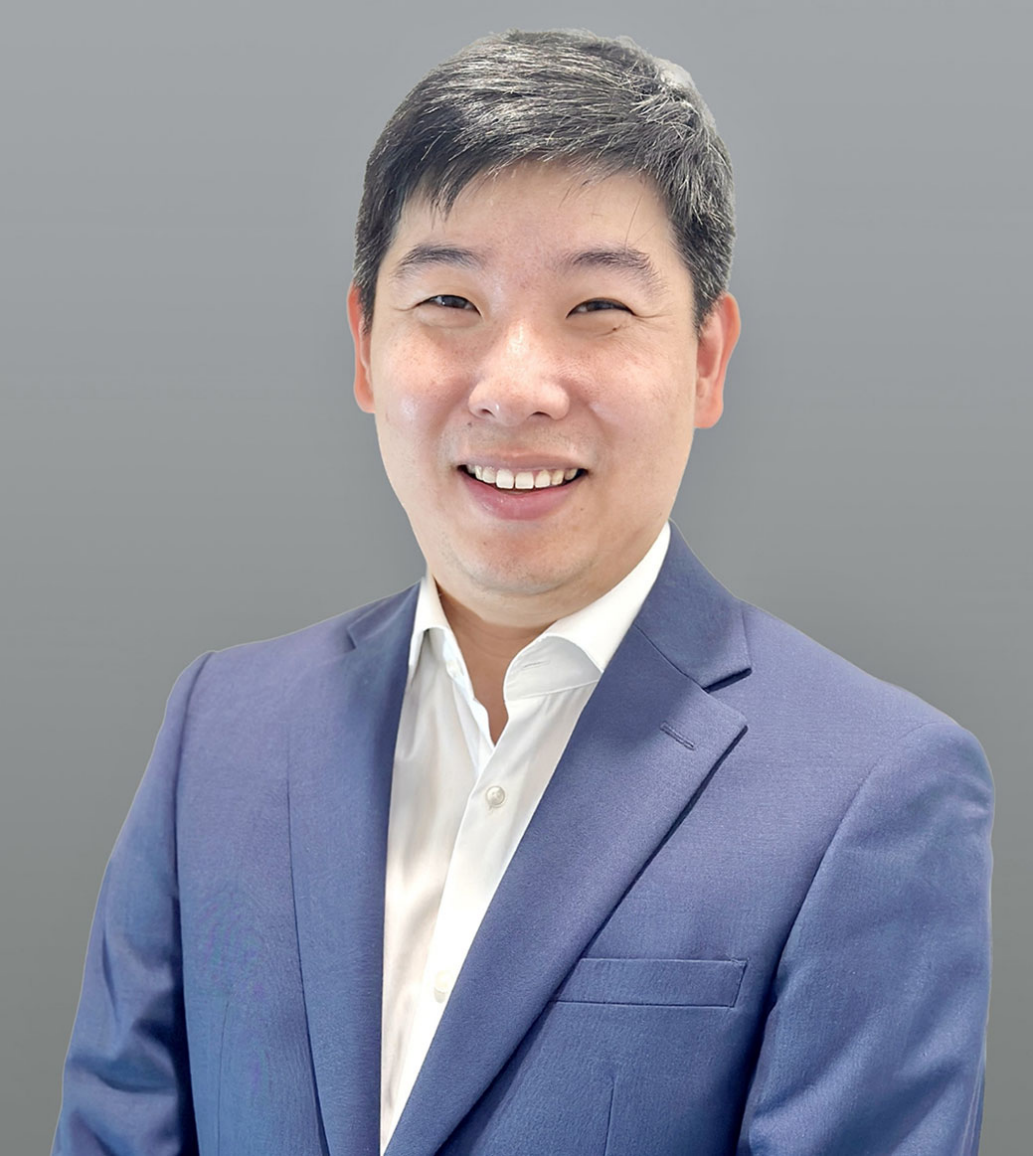


**Jun-yi Zhu**

**How would you explain the main findings of your paper to non-scientific family and friends?**

African Americans have a higher risk of developing kidney diseases because most of them carry the APOL1 risk alleles G1 or G2. However, the molecular mechanisms of how these two risk alleles G1 and G2 cause kidney diseases are still unknown. We have generated a fly kidney disease model that is induced by expressing APOL1 risk alleles G1 or G2. We found that in fly kidney cells/nephrocytes, expression of APOL1 risk alleles G1 or G2 affects cell function and causes cell death. We further identified that these phenotypes are due to the defects of cell autophagy. Our findings suggest new therapeutic targets to treat APOL1-induced kidney diseases.

**What are the potential implications of these results for your field of research?**

Currently, more and more attention has been paid to the field of APOL1-induced kidney diseases. However, there is no treatment for it. Understanding the molecular mechanisms and providing drug treatment targets is fundamentally important to many future APOL1-induced kidney disease studies. Our study provides the possible molecular mechanisms of how the APOL1 risk alleles G1 and G2 cause kidney diseases, and a potential new therapeutic target is suggested in our study. Therefore, we hope that this research is followed by other scientists interested in APOL1-induced kidney diseases.

[…] autophagy pathways are severely affected by the expressed APOL1 risk alleles.

**Figure:**
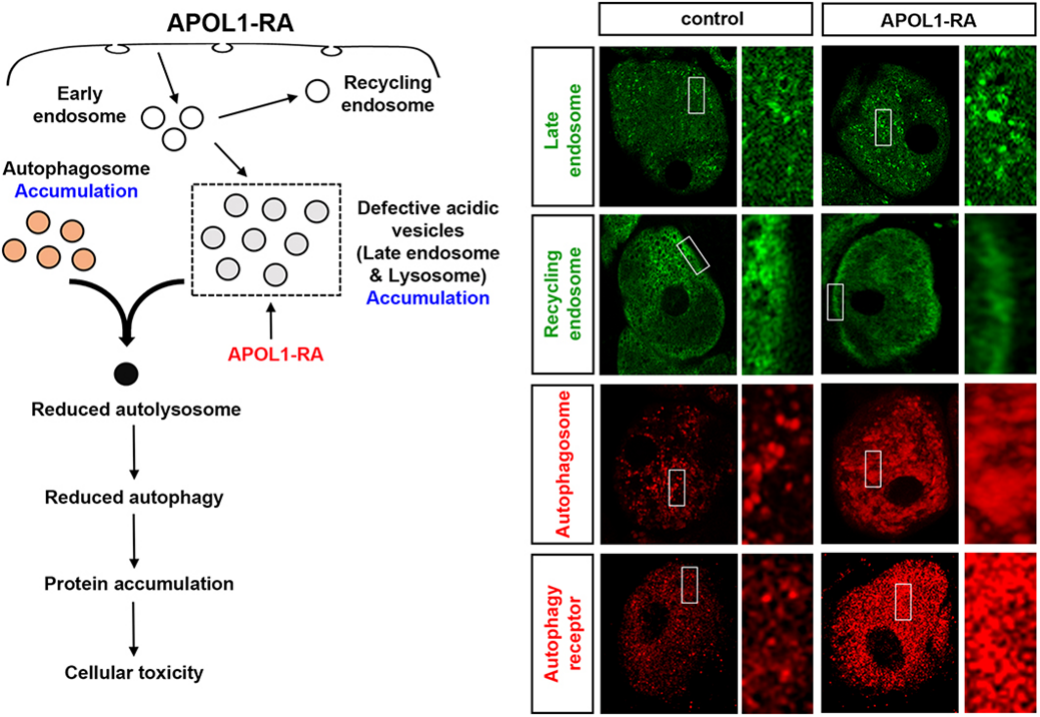
Model of APOL1-RA mediated disruption of the autophagy pathway.

**What has surprised you the most while conducting your research?**

There is no clear mechanism and therapeutic targets to treat APOL1-induced kidney diseases have been identified previously. The fly is an important model for studying organ development and disease due to the remarkable conservation in gene function across species and, although fly kidney cell bears striking structural and functional similarities to the mammalian kidney cell podocyte, we were slightly worried that we could not identify the molecular mechanisms on how APOL1 risk alleles affect cell function and cause cell death. It is especially exciting to find that autophagy pathways are severely affected by the expressed APOL1 risk alleles. Through additional assays to support these findings, we identified the potential molecular mechanisms of APOL1 risk alleles causing cell death and inducing kidney diseases.

**Describe what you think is the most significant challenge impacting your research at this time and how will this be addressed over the next 10 years?**

For most human diseases, we still have less knowledge about the disease's cause. We do not have clear clues regarding the function of the disease-causing genes and why the mutation of a gene will cause human diseases. It will be critical to understand the functions of genes in both normal and diseased conditions. The fly model can provide a cost-effective and fast platform for these purposes.

**What's next for you?**

In the current study, we identified the molecular mechanisms of APOL1 risk alleles causing kidney disease and provide potential therapeutic targets to treat APOL1-induced kidney diseases. However, there is still no effective treatment for this APOL1-induced kidney disease. Next, I would like to test different drugs targeting autophagy pathways, which we identified in this study to treat flies expressing APOL1 risk alleles G1 and G2. A fly can be a great model to test different drugs easily and quickly. Once the drugs have been identified in flies that can rescue APOL1-induced kidney diseases, we will validate them again in cultured human cells. This will provide preclinical evidence of the potential drugs targeting autophagy to treat APOL1-induced kidney diseases.

## References

[DMM050591C1] Zhu, J.-y., Lee, J.-G., Fu, Y., van de Leemput, J., Ray, P. E. and Han, Z. (2023). *APOL1-G2* accelerates nephrocyte cell death by inhibiting the autophagy pathway. *Dis. Model. Mech.* 16, dmm050223. 10.1242/dmm.050223 37969018 PMC10765414

